# STAT3 Oligonucleotide Inhibits Tumor Angiogenesis in Preclinical Models of Squamous Cell Carcinoma

**DOI:** 10.1371/journal.pone.0081819

**Published:** 2014-01-03

**Authors:** Jonah D. Klein, Daisuke Sano, Malabika Sen, Jeffrey N. Myers, Jennifer R. Grandis, Seungwon Kim

**Affiliations:** 1 Department of Otolaryngology, School of Medicine, University of Pittsburgh, Pennsylvania, United States of America; 2 Department of Biology and Function in Head and Neck, Yokohama City University Graduate School of Medicine, Yokohama, Japan; 3 Department of Head and Neck Surgery, University of Texas M.D. Anderson Cancer Center, Texas, United States of America; 4 Department of Pharmacology and Chemical Biology, School of Medicine, University of Pittsburgh, Pennsylvania, United States of America; Jawaharlal Nehru University, India

## Abstract

**Purpose:**

Signal transducer and activator of transcription 3 (STAT3) has shown to play a critical role in head and neck squamous cell carcinoma (HNSCC) and we have recently completed clinical trials of STAT3 decoy oligonucleotide in patients with recurrent or metastatic HNSCC. However, there is limited understanding of the role of STAT3 in modulating other aspects of tumorigenesis such as angiogenesis. In this study, we aimed to examine the effects of STAT3 decoy oligonucleotide on tumor angiogenesis.

**Experimental Design:**

A STAT3 decoy oligonucleotide and small interfering RNA (siRNA) were used to inhibit STAT3 in endothelial cells *in vitro* and *in vivo*. The biochemical effects of STAT3 inhibition were examined in conjunction with the consequences on proliferation, migration, apoptotic staining, and tubule formation. Additionally, we assessed the effects of STAT3 inhibition on tumor angiogenesis using murine xenograft models.

**Results:**

STAT3 decoy oligonucleotide decreased proliferation, induces apoptosis, decreased migration, and decreased tubule formation of endothelial cells *in vitro*. The STAT3 decoy oligonucleotide also inhibited tumor angiogenesis in murine tumor xenografts. Lastly, our data suggest that the antiangiogenic effects of STAT3 decoy oligonucleotide were mediatedthrough the inhibition of both STAT3 and STAT1.

**Conclusions:**

The STAT3 decoy oligonucleotidewas found to be an effective antiangiogenic agent, which is likely to contribute to the overall antitumor effects of this agent in solid tumors.Taken together with the previously demonstrated antitumor activity of this agent, STAT3 decoy oligonucleotide represents a promising single agent approach to targeting both the tumor and vascular compartments in various malignancies.

## Introduction

Signal transducer and activator of transcription 3 (STAT3) has been proven to be critical in tumor cell growth, viability, and metastasis in head and neck cancer among other cancer types [1]. STAT3 is activated downstream of both the EGFR and IL6 signaling pathways. Both EGFR expression and IL6 production are poor prognostic indicators for many cancers and therapeutic agents that target these pathways are either established treatments or are under investigation for many malignancies including HNSCC [2], [3]. Due to the involvement of STAT3 in key regulatory pathways in tumor cells, several investigators have examined the efficacy of inhibiting STAT3 as anti-cancer therapy. One promising approach that has been used to inhibit STAT3 is the decoy oligonucleotide strategy. The STAT3 decoy oligonucleotide is a 15-mer double stranded oligonucleotide that mimics the STAT3 binding region on the endogenous DNA, acting primarily by competing with the endogenous DNA for binding of activated STAT3 dimers. The STAT3 decoy oligonucleotide has been studied as anti-tumor agent in several types of cancer such as ovarian, breast, hepatocellular, and lung cancer as well as malignant glioma [4]–[8]. We have shown previously that the STAT3 decoy oligonucleotide is able to inhibit the growth of HNSCC cell lines and xenografts in nude mice [9]–[12]. Based primarily on our promising preclinical data, we have recently completed a phase 0, biomarker driven clinical trial of STAT3 decoy oligonucleotide in patients with resectable HNSCC [13]. The results of this study was recently published and demonstrated that STAT3 decoy oligonucleotide is able to efficiently downregulate STAT3 regulated genes in human tumors.

Given the promise of the STAT3 decoy oligonucleotide as a therapeutic approach in HNSCC, we aimed to fully understand the mechanisms by which the STAT3 decoy oligonucleotide exerts its antitumor effects. It is well established that STAT3 is activated in endothelial cells by various cytokines such as IL-6 and vascular endothelial growth factor (VEGF) [14]–[16]. STAT3 is believed to play a critical role in the physiology of endothelial cells including responses to oxidative stress and inflammation [17], [18]. Therefore, we hypothesized thatSTAT3 decoy oligonucleotide is an efficient inhibitor of tumor angiogenesis. By utilizing *in vitro* assays as well as scaffold-based *in vivo* angiogenesis assay, we show that the STAT3 decoy oligonucleotide has potent, direct antiangiogenic properties. These results underscore the utility of STAT3 decoy oligonucleotide as a single agent that targets both the tumor cell and endothelial cell compartment and provide further rationale for the development of STAT3 inhibitors for treatment of solid malignancies.

## Materials and Methods

### Cell lines

Human umbilical vein endothelial cells (HUVEC) and human dermal microvascular endothelial cells (HDMEC) were our endothelial cell models (Lonza, Walkersville, MD). These endothelial cells were grown in microvascular endothelial cell growth medium-2 (Lonza, Walkersville, MD). T24 cell line was maintained in DMEM (Mediatech, Manassas, V) and 10% FBS (Bioexpress, Kaysville, UT).

### Reagents

The STAT3 decoy oligonucleotide sequence was 5′-CATTTCCCGTAAATC-3′, 3′GTAAAGGGCATTTAC-5′ and the STAT3 mutant control oligonucleotide was 5′-CATTTCCCTTAAATC-3′, 3′-GTAAAGGGAATTTAG-5 and the first and last three bases in each strand were phosphorothioated (IDT, San Diego, CA). We also used a sense strand that was tagged at the 5-end with Fluorescein amidite (FAM). The decoy oligonucleotide was annealed prior to use by boiling for 5 minutes and made to 100 µM stock. The STAT3 decoy oligonucleotide used *in vivo* (NSC-741763) is the identical sequence, but double stranded (NCI, Biopharmaceutical Development Program, Frederick, MD) [12]. The human pooled STAT1 (L-003543-00) and STAT3 (M-003544-02) specific siRNA was used to downregulate STAT1 and STAT3 *in vitro* (Dharmacon Products, Lafayette, CO). The following antibodies were used for immunohistochemical analysis: rat monoclonal anti-CD31/platelet/endothelial cell adhesion molecule 1 (CD31/PECAM-1) (PharMingen, SanDiego, CA); peroxidase-conjugated goat anti-rat immunoglobulin G1 (Jackson Research Laboratories, West Grove, PA) and Alexa Fluor 594–conjugated goat anti-rat immunoglobulin G (Invitrogen, Carlsbad, CA).

### Cell viability assay

HUVECs or HDMECs were plated at 3×10^4^ cells per well in a 24 well plate and grown in complete EGM-2 media. After 24 hours the cells were transfected with the STAT3 decoy oligonucleotide (0 nM–10000 nM) and a mutant control oligonucleotide (10000 nM) using Lipofectamine 2000 (Invitrogen, Carlsbad, CA) in optimum growth medium (Invitrogen, Carlsbad, CA). Following transfection the cells were placed in complete EGM-2. 72 hours post treatment the cells were assessed for viability using Thiazolyl Blue Tetrazolium Bromide (MTT) (Sigma, St. Louis, MO).

### Apoptosis assay

1×10^6^ HUVECs were plated on 10 cm dishes and grown in complete EGM-2. After 24 hours the cells were transfected with the STAT3 decoy oligonucleotide (0 nM–100 nM) or mutant control oligonucleotide (100 nM), or treated with cisplatin as positive control. After 24 hours the cells were trypsinized, washed, and resuspended in 1× binding buffer (BioVision, San Francisco, CA). FITC-conjugated Annexin-V reagent (BioVision, San Francisco, CA) was added to the cells and the cells were analyzed with a FACScalibur (BD Biosciences, San Diego, CA) to quantify the number of cells positive for FITC. The data was represented as the percentage of cells positive for Annexin-V detection.

### In vitro tubule formation assay

HUVECs were transfected with either the STAT3 decoy oligonucleotide (100 nM) or the STAT3 mutant control oligonucleotide (100 nM) using lipofectamine 2000 in optimum growth medium. 24 hours post transfection, 5×10^3^ treated HUVECs were plated in a 96 well plate on top of growth factor reduced Matrigel (BD Biosciences, San Diego, CA). After 24 hours the wells were imaged using 4× phase contrast microscopy and the images were quantified for tubule formation.

### Live cell imaging

5×10^5^ HUVECs were plated in a 10 cm dish and transfected with either the STAT3 mutant control oligonucleotide or the FAM-tagged STAT3 decoy oligonucleotide. Twenty four hours after transfection, 5×10^4^ HUVECs were plated on top of Matrigel in a 0.17 mm clear Delta T Dish (BioptechsInc, Butler, PA). The cells were placed in 37°C for 15 minutes to allow adherence and were subsequently imaged on a Nikon Ti inverted microscope with perfect focus using phase contrast at 10×. Images were captured every 10 minutes for 24 hours in 8 unique fields with a Roper Scientific Cascade 1K camera using MetaMorph 7.73 software. Cell tracking was performed with the track objects application in Metamorph to obtain mean distance traveled, mean velocity and distance from origin.

### Transwell migration assay

HUVECs were transfected with either the STAT3 decoy (100 nM) or the STAT3 mutant control (100 nM) using lipofectamine 2000 in optimum growth medium. 24 hours post transfection, 1×10^3^ treated HUVECs were plated in a hydrated 8.0 micron control insert (BD Biosciences, San Diego, CA) with serum free EBM-2 (Lonza, Walkersville, MD) and placed in a 24-well companion plate containing complete EGM-2. After 24 hours the inserts were fixed and stained using the Protocol Hema 3 Stain Set (Fisher Scientific Company, Kalamazoo, MI). The cell number in each insert was counted and recorded as number of cells migrated.

### Xenograft formation and treatment

All animal studies were performed with approval by the Institutional Animal Care and Use Committee at the University of Pittsburgh (approval number 0909400). 2×10^6^UM-22B HNSCC cells were injected subcutaneouslyinto flanksof nude mice. Once palpable tumors were achieved, treatment was initiated with the STAT3 decoy oligonucleotide (50 µg/50 µl), the STAT3 mutant control oligonucleotide (50 µg/50 µl), or the vehicle control (50 µl PBS) via intratumoral injection. The tumors were treated for three weeks. At the end of the treatment period, the mice were sacrificed. The tumor xenografts were harvested and frozen at −80°C.

### Immunohistochemical-Immunofluorescent Analysis

Frozen tissues were used for detection of CD31/PECAM-1, and terminal deoxynucleotidyltransferase-mediated dUTP nick end labeling (TUNEL). Slides were prepared as previously described [19]. Immunostaining for CD31/PECAM-1 (1∶400) were performed using the methods indicated as previously described [19]. TUNEL assay was performed using DeadEndFluorometric TUNEL System (Promega, Madison, WI). For CD31-TUNEL double staining, TUNEL staining was performed on slides already labeled with anti-CD31 antibody, as described above. Endothelial cells were identified by red fluorescent staining, and DNA fragmentation was detected by localized green and yellow fluorescence within the nuclei of apoptotic cells.

### Quantification of Microvessel Density, Apoptotic Tumor and Endothelial Cells

For quantification of TUNEL expression, the cells positively stained were counted in 15 0.04-mm^2^ fields with a 20× objective.Vessels completely stained with anti-CD31 antibodies were counted in random 0.04-mm^2^ fields at using a 20× objective. Quantification of apoptotic endothelial cells (CD31/TUNEL) was measured as the average of the ratio of apoptotic endothelial cells to the total number of endothelial cells in 10 random 0.01-mm^2^ fields at using a 40× objective.

### In vivo angiogenesis assay using PLLA scaffolds

Poly-L-lactic acid (PLLA) scaffolds were prepared as previously described and cut into 6 mm×6 mm×1 mm sections [20], [21]. 1×10^6^ HUVECs per sponge were resuspended 1∶1 with media and Matrigel (BD Biosciences, San Diego, CA). Each scaffold was seeded with the 1×10^6^ endothelial cells and incubated at 37°C for 30 minutes and then placed on ice. The sponges were implanted 2 per mouse on both flanks via subcutaneous implantation as previously described [20], [21]. After 3 days in the mice, the mice were treated via per-scaffold injections of either the clinical grade STAT3 decoy oligonucleotide (50 µg/50 µl) or the STAT3 mutant control oligonucleotide (50 µg/50 µl) for 15 days. At the end of the treatment period, the scaffolds were excised, formalin fixed and paraffin embedded.

### Western blotting

40 µg of protein was resolved in an 8% SDS-PAGE gel and transferred onto a Nitrocellulose membrane using a semidry transfer machine (Bio-Rad Laboratories, Hercules, CA). Western blot was then performed with the appropriate primary and secondary antibodies. The blot was developed with luminol reagent (Santa Cruz Biotechnology, Santa Cruz, CA). The band intensity was quantitated with DigiDoc1000 software (Alpha Innatech Corporation, San Leandro, CA).

### Statistical analysis

The quantifications were compared with the paired Student's*t*-test. Statistical analyses were performed with Prism 5.01 software (GraphPad Software). *P* values<0.05 were considered statistically significant.

## Results

### STAT3 decoy oligonucleotide decreases cell viability and induces apoptosis of human endothelial cells

**Figure 1:**
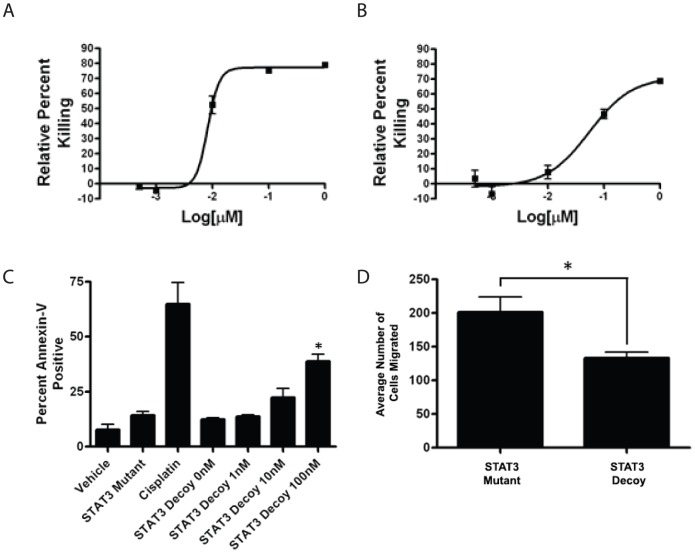
Effects of STAT3 decoy oligonucleotide on the proliferation, apoptosis, and migration of HUVECs and HDMECs*in vitro*. (A) HUVECs and (B) HDMECs were transfected with the STAT3 decoy oligonucleotide or mutant control oligonucleotide at 0 nM to 1000 nM. Seventy two hours later the cells were assessed for proliferation using MTT assay. Results are shown in percent kill relative to the mutant control oligonucleotide and the experiments were performed in triplicate. (C) HUVECs were transfected with the either STAT3 decoy oligonucleotide (0 nM to 100 nM), the mutant control oligonucleotide (100 nM), vehicle, or cisplatin (100 µM). Twenty four hours later the cells were analyzed for apoptosis using Annexin V staining and flow cytometry. The experiments were performed in triplicate (* p<0.05). (D) HUVECs were transfected with 100 nM of STAT3 decoy oligonucleotide or 100 nM of STAT3 of mutant control oligonucleotide, plated inTranswell8uM pore inserts, and extent of migration was ascertained 24 hrs later. STAT3 decoy oligonucleotide decreased the migration of HUVEC compared with the mutant control oligonucleotide (* p<0.05). All of the experiments were performed in triplicate.

We first assessed the *in vitro* effects of the STAT3 decoy oligonucleotide on endothelial cells by examining the effects of STAT3 decoy on the viability of HUVEC and HDMEC (Fig. 1A & B). Compared with the mutant control oligonucleotide, STAT3 decoy oligonucleotide treatment induced a dose-dependent decrease in the viability of HUVEC and HDMEC. The effective IC_50_ of STAT3 decoy treatment for HUVEC and HDMEC were 8 nM and 137 nM, respectively. Both the HUVEC and HDMEC demonstrated greater sensitivity to the STAT3 decoy oligonucleotide than any of HNSCC cell lines tested in previously published studies, highlighting the importance of STAT3 in endothelial cell physiology and angiogenesis [9]. While both HUVEC and HDMEC were sensitive to the STAT3 decoy oligonucleotide, HUVEC was used as the primary model for the subsequent experiments as its sensitivity to the STAT3 decoy was higher than HDMEC.

It is well known that some of the STAT3 target genes include genes such as Bcl-xl which is involved in antiapoptosis [22]. Therefore, we next examined the effects of STAT3 decoy oligonucleotide on the apoptosis of HUVEC. HUVECs were treated with increasing concentrations of STAT3 decoy oligonucleotide and the degree of apoptosis was examined using a flow cytometric apoptosis assay. Compared to the mutant control oligonucleotide, STAT3 decoy oligonucleotide produced a dose-dependent induction of apoptosis in HUVEC (Fig. 1C). Lastly, we examined the effects of STAT3 oligonucleotide on the migration of HUVEC using trans-well migration assay. In this assay, STAT3 decoy oligonucleotide decreased the migration of endothelial cells when compared to the STAT3 mutant control oligonucleotide (p<0.05) (Fig. 1D).

### STAT3 decoy inhibits HUVEC tubule formation

**Figure 2:**
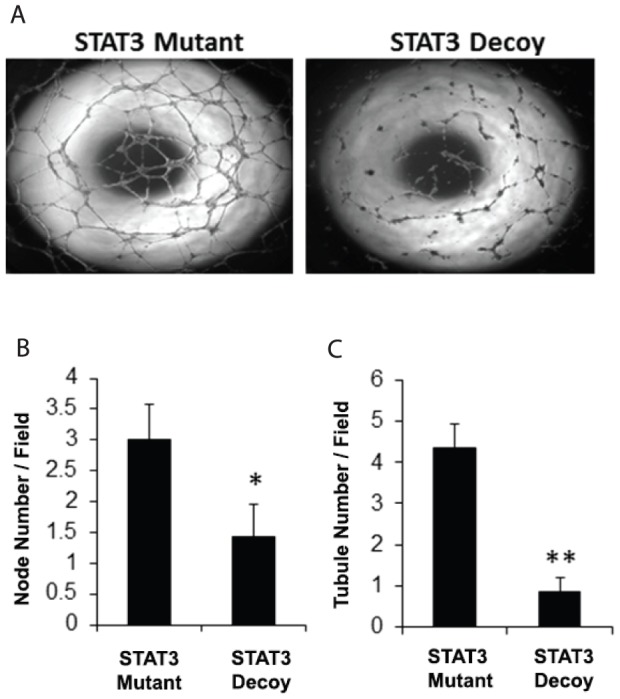
STAT3 decoy inhibits HUVEC tubule formation. (A) HUVECs were transfected with 100 nM of STAT3 decoy oligonucleotide or 100 nM of STAT3 mutant control oligonucleotide. Twenty four hours later 15,000 cells were plated on top of Matrigel in a 96-well plate. After 24 additional hours the plates were assessed for tubule formation. The tubule formation was the quantified by counting the total number of (B) nodes and (C) tubules per field. Stat3 decoy oligonucleotide decreased both node and tubule number compared to the STAT3 mutant (*, ** p<0.05). All of the experiments were performed in triplicates.

We next examine the *in vitro* effect of the STAT3 decoy oligonucleotide on the angiogenic potential of HUVECs using tubule formation assay. HUVECs were treated with either the STAT3 decoy oligonucleotide or the mutant control oligonucleotide and then plated on Matrigel. Compared with the mutant control oligonucleotide, STAT3 decoy oligonucleotide produced a significant decrease in tubule formation (Fig. 2A). The degree of tubule formation was quantified by assessing the number of tubules and the number of nodes (intersection point of at least two tubules). Both tubule node and tubule number were significantly decreased with STAT3 decoy oligonucleotide treatment compared with the mutant control oligonucleotide (p<0.05)(Fig. 2B & C).

**Figure 3:**
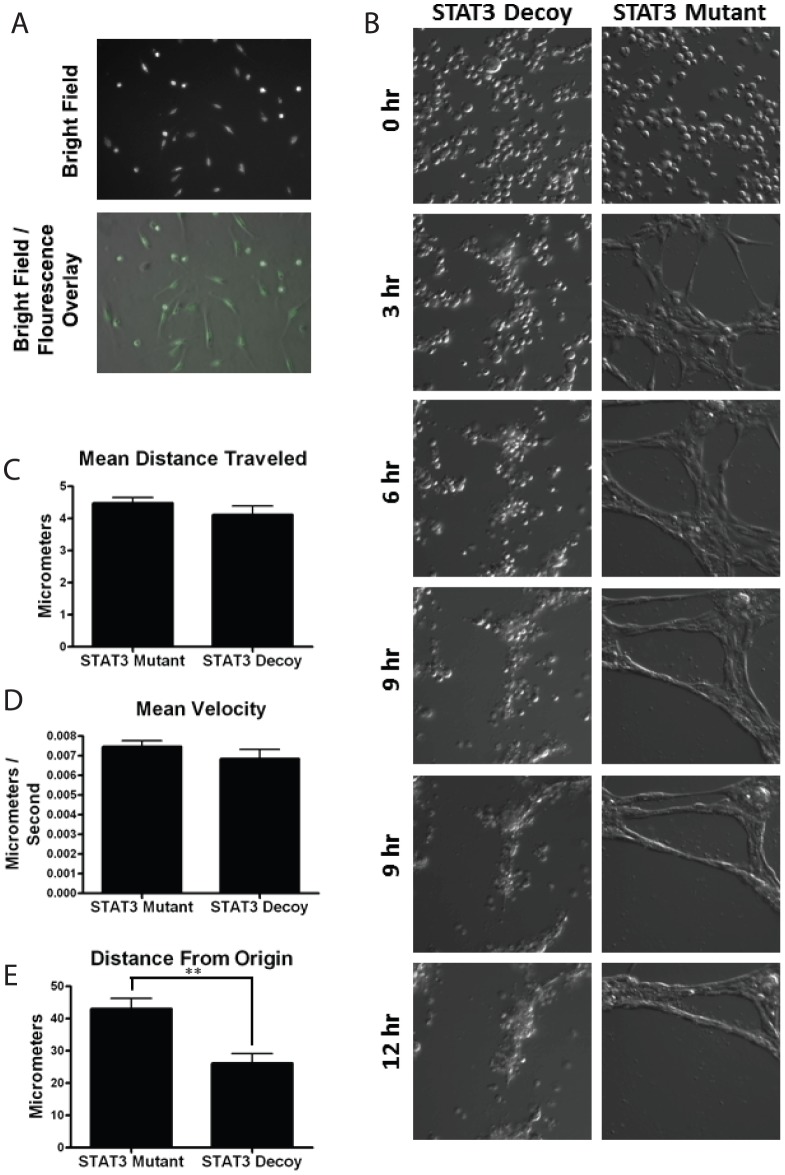
STAT3 decoy inhibits net migration of HUVECs during *in vitro* tubule formation. HUVECs were transfected with 100× every ten minutes for the following 24 hours in eight different fields. (A) The decoy was FAM-tagged (green) in order to ensure positive transfection. (B) Representative images from various time points from the course of the experiment.The live cell images were quantified for three distinct parameters: (C) mean distance traveled,(D) mean velocity, and (E) distance from origin. The distance from origin was significantly decreasedby STAT3 decoy oligonucleotide compared to the STAT3 mutant oligonucleotide (** p<0.05). All of the experiments were performed in triplicates.

In order to further characterize the effects of STAT3 decoy oligonucleotide on the process of tubule formation, we performed a live-cell assay in which the process of tubule formation was examined with serial photography. HUVECs treated with the STAT3 decoy oligonucleotide or mutant control oligonucleotides were plated on Matrigel and images were taken every 10 minutes for 24 hours in 8 unique fields of each treatment. The STAT3 decoy oligonucleotides were FAM-tagged to confirm successful transfection (Fig. 3A). Using computer-assisted analysis, all HUVECs that remained in the field the duration of the experiment were tracked (Fig. 3B). This novel live assay allowed us to assess specific movement parameters during the process of tubule formation including mean distanced traveled, mean velocity and mean distance from origin (Fig. 3C–E). Despite the decrease in tubule formation with STAT3 decoy oligonucleotide treatment, there were no statistically significant differences in the mean distance traveled and the mean velocity between the mutant control and STAT3 decoy oligonucleotide treated HUVECs. However, there was a statistically significant decrease in the mean distance traveled from the point of origin in HUVECs treated with STAT3 decoy oligonucleotide compared with HUVECs treated with STAT3 mutant control oligonucleotide (p<0.05). Although this data is preliminary, it suggests that the inhibition of tubule formation by STAT3 decoy oligonucleotide may occur by interference with the chemotactic rather than the migratory aspect of tubule formation.

### STAT3 decoy oligonucleotide inhibits tubule formation in vitro via inhibition of both STAT1 and STAT3

**Figure 4:**
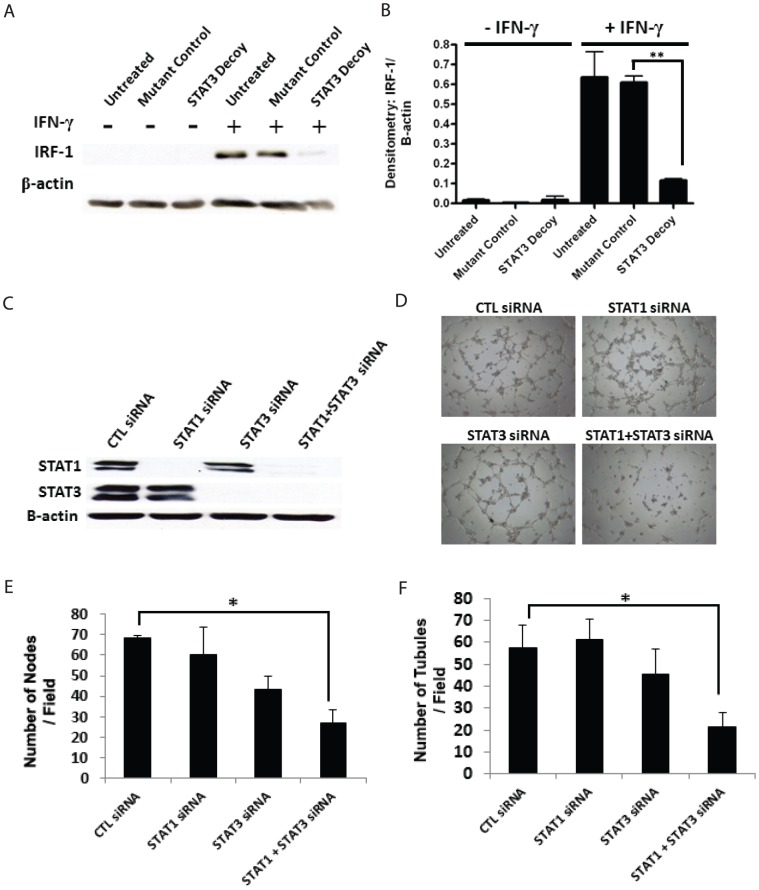
STAT3 decoy has additionally activity against STAT1. (A) HUVECs were transfected with 100 nM of STAT3 decoy oligonucleotide. Twenty four hour after transfection, the cells were stimulated with IFN-γ (20 ng/ml for one hr) and Western blot was performed for IRF-1. (B) Densitometric measurement of IRF-1 (** P<0.05). (C) HUVECs were transfected with either STAT1 siRNA, STAT3 siRNA or both. Western blot was then performed to confirm the downregulation of STAT1, STAT3, or both. (D) Downregulation of STAT1 or STAT3 did not result in decreased tubule formation. In contrast, combined downregulation of both STAT1 and STAT3 resulted in statistically significant decrease in tubule formation. Quantification of tubule formation by (E) total number of nodes per field and (F) total number of tubules per field (* p<0.05).

While the STAT3 decoy oligonucleotide is effective at abrogating STAT3-mediated gene transcription, previous work from our lab demonstrated that it can also binds to STAT1 [23]. We therefore sought to examine whether the STAT3 decoy oligonucleotide inhibited STAT1 in endothelial cells. HUVECs treated with the STAT3 decoy oligonucleotide or mutant control decoy were stimulated with interferon gamma (IFN-γ), and Western blot was then performed for interferon response factor 1 (IRF-1). Induction of IRF-1 expression by IFN-γ is primarily a STAT1 mediated process, and serves as an indicator of the integrity of the STAT1 pathway [24]. We observed that treatment of HUVEC with STAT3 decoy oligonucleotide inhibited the IFN-γ induced IRF-1 (Fig. 4A & B). This data suggested that in HUVEC, STAT3 decoy oligonucleotide binds to not only STAT3 but STAT1 as well.

To examine whether the effects of STAT3 oligonucleotide on tubule formation was due to the inhibition of STAT3, STAT1 or both, we then examined the effects of the downregulation of STAT1 alone, STAT3 alone, or both STAT1 and STAT3 on HUVEC proliferation and tubule formation. The down regulation of STAT1 alone or STAT3 alone did not result in statistically significant decrease in tubule formation compared with the control siRNA. However, only the combined downregulation of STAT1 and STAT3 resulted in statistically significantdecrease in tubule formation compared with the control siRNA (Fig. 4C–F). These results suggest that both STAT1 and STAT3 are involved in the process of *in vitro* tubule formation and that the decrease in tubule formation seen with STAT3 decoy oligonucleotide is produced by the dual inhibition of STAT1 and STAT3.

### Treatment of tumor xenografts with STAT3 decoy oligonucleotide decreases microvessel density in vivo

**Figure 5:**
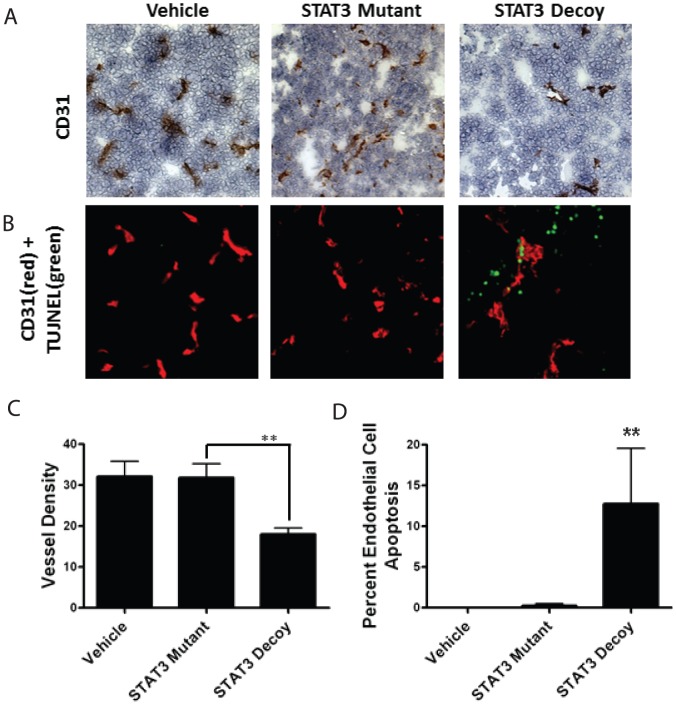
STAT3 decoy treatment of tumor xenografts decreases microvessel density and increases endothelial cell apoptosis. HNSCC xenografts were produced in nude mice using the cell line UM-22B. The mice were then treated with direct intratumoral injection with vehicle, STAT3 decoy, or mutant control oligonucleotide for 3 weeks. The tumors were then analyzed with immunohistochemical staining for CD31 for microvessel density or dual staining for CD31/TUNEL for endothelial cell apoptosis. Representative images of (A) CD31 stainingand (B) dual CD31/TUNEL staining, where red is CD31 and green is TUNEL. (C) Quantification of microvessel density (** p<0.001). (D) Quantification endothelial cell apoptosis density (** p<0.001).

While the HUVECs are one of the most frequently utilized cell-model for studying angiogenesis *in vitro*, tubule formation assays do not replicate the complex process of *in vivo* tumor angiogenesis. In order to confirm our *in vitro* findings, we examined the *in vivo* ability of STAT3 decoy oligonucleotide to inhibit tumor angiogenesis. For this study, HNSCC cell line UM-22 was used to produce xenografts in nude mice. Once palpable tumors were present, the mice were treated daily with intratumoral injection of vehicle (saline), mutant control oligonucleotide, or the STAT3 decoy oligonucleotide. The tumors were then examined with both CD31 immunohistochemistry and dual CD31/TUNEL immunofluorescence (Fig. 5A & B). Compared with the mutant control oligonucleotide treated or saline treated tumors, STAT3 decoy oligonucleotide treated tumors showed a statistically significant decrease in microvessel density (p<0.05) (Fig. 5a and 5b). Additionally, dual CD31/TUNEL staining showed an increase in the endothelial cell apoptosis in the STAT3 decoy oligonucleotide group compared to the mutant control oligonucleotide or saline treated group (p<0.05) (Fig. 5C & D).

### STAT3 decoy oligonucleotide directly inhibits angiogenesis

**Figure 6:**
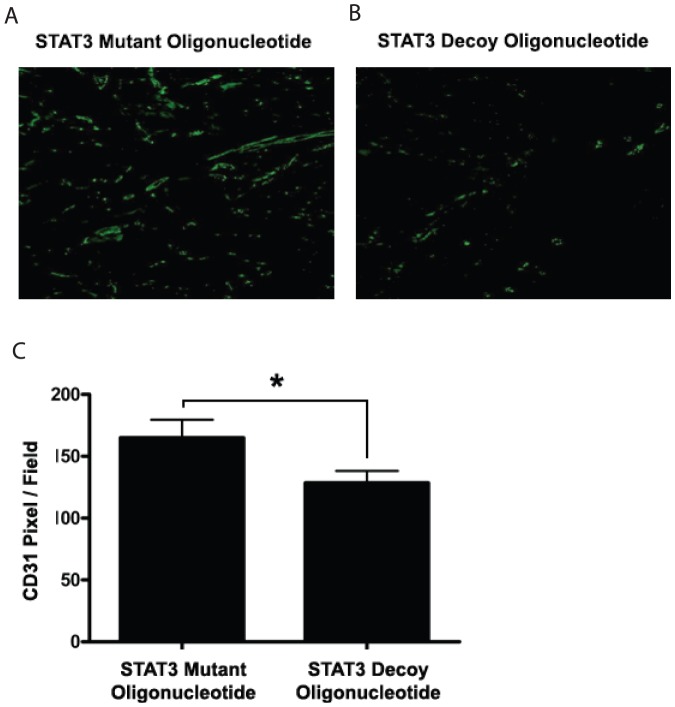
STAT3 decoy decreases microvessel density *in vivo* by acting directly on the endothelial cells. Ploy-L-lactic acid scaffolds were seeded with HUVECs and then implanted on the flank of nude athymic mice. The mice were the treated with either the STAT3 decoy oligonucleotide or the STAT3 mutant control via peri-scaffold injection for 15 days. (A&B)) Representative images from CD31 staining of scaffolds treated with either STAT3 decoy of STAT3 mutant control showing decreased microvessel density with STAT3 decoy oligonucleotide. (C) Quantification of microvessel density in both treatment groupsshowed decreased microvessel density in STAT3 decoy oligonucleotide treated group (*p<0.05).

It has been well documented that VEGF is a STAT3 target gene and that the inhibition of STAT3 results in decreased production of VEGF [25]–[27]. We next sought to examine whether the decrease in the microvessel density (seen in the previous experiment) was due to a direct effect of the STAT3 decoy oligonucleotide on the endothelial cell or a result of a decrease in tumor cell production of VEGF. We utilized, for this purpose, a murine model of *in vivo* angiogenesis that is not dependent on the presence of any tumor cells. In this assay, HUVECs and Matrigel (1∶1) were seeded onto poly-L-lactic acid (PLLA) sponges and then the sponges were implanted into subcutaneous tissue of nude mice [20], [21]. The mice were then treated via peri-scaffold injection of 50 µg of either the STAT3 decoy oligonucleotide or the mutant control oligonucleotide. The sponges were removed after two weeks of treatment and immunofluorescent staining was performed against human CD31. Treatment of the PLLA sponges with STAT3 decoy oligonucleotide resulted in a statistically significant decrease in the formation of blood vessels within the sponges compared with PLLA sponges treated with STAT3 mutant decoy oligonucleotide (p<0.05) (Fig. 6A–C). These results suggested that the decreases in microvessel density seen in tumor xenograft were in part due to direct effect of the STAT3 decoy oligonucleotide on tumor endothelium.

## Discussion

STAT3 has been proposed as a therapeutic target for tumor cells as it is a downstream mediator of EGFR and IL6, both of which have been shown to be important in carcinogenesis and angiogenesis [28]. There are small molecule tyrosine kinase inhibitors that have been designed to inhibit the janus kinase 2 (JAK2)-STAT3 pathway including AG490 and AZD1480, and several of these agents are being studied in clinical trial setting [29], [30]. We have been interested in the use of decoy oligonucleotide as a therapeutic approach for inhibiting STAT3. Our institution has recently initiated and completed a biomarker-driven neoadjuvant phase 0 clinical trial of the STAT3 decoy in patients with resectable head and neck cancer. In fact, the STAT3 decoy oligonucleotide is the only STAT3 inhibitor that has progressed to clinical evaluation in patients with cancer of thehead and neck [11]. Despite the progress of the STAT3 decoy as a therapeutic agent in head and neck cancer, little work has been done to understand how this agent affects other aspects of tumorigenesis such as angiogenesis. Additionally, there has never been an agent designed to inhibit angiogenesis by targeting nuclear transcription factors.

We have demonstrated in this study that the STAT3 decoy oligonucleotide has significant antiangiogenic properties. We have found that STAT3 decoy oligonucleotide decreased the viability and induced apoptosis of HUVECs and HDMECs *in vitro*. It should be noted that STAT3 decoy oligonucleotide does not have cytotoxic effects on normal tissues such as normal oral keratinocytes in the range used in our study [9].

The role of STAT1 and STAT3 in the process of angiogenesis has been examined in several previous publications. It has been shown in several studies that STAT3 is downstream from VEGFR2 and is activated by the binding of VEGF to VEGFR2 [15], [16], [31]. Schaefer et al has found that STAT3 is constitutively activated in tumor endothelium of glioma and medulloblastoma tumors suggesting that STAT3 is involved in tumor angiogenesis [32]. On the other hand, several studies have suggested that STAT1 is a negative regulator of angiogenesis. A study by Battle et al showed that the activation of STAT1 by IFN-γ lead to decreased tubule formation by HUVEC *in vitro*. This study further found decreased microvessel density in STAT1-/- mice with Matrigel plug assay compared with wild-type control mice [33]. The role of STAT1 in tumor angiogenesis was assessed in a study by Huang et al using a highly angiogenic murine fibrosarcoma cell line derived from STAT1 -/- mice. When STAT1 expression was restored using a STAT1 expression vector, the cell line showed decreased growth *in vivo* as well as decreased microvessel density [34]. Our data suggest that the inhibitory effect of STAT3 decoy oligonucleotide on angiogenesis may be due to the inhibition of both STAT1 and STAT3. One possible explanation for this effect is that the STAT1/STAT3 heterodimers have a distinct role in tumor angiogenesis independent from the role of STAT1 or STAT3 homodimers, but further work is necessary to elucidate this effect.

While we are convinced that the anti-angiogenic effect we observe with the STAT3 decoy is not solely due to its inhibition of STAT3, the agent is nonetheless effective at inhibiting tubule formation *in vitro* and in decreasing tumor angiogenesis *in vivo*. The goal of this study was to identify a compound that can effectively target both the tumor cell and endothelial cell compartment of a tumor. Other such therapeutic approaches include strategies such as combining Bevacizumab with EGFR TKIs such as Erlotinib [35]. However, one limitation of a dual agent approach is the increase in adverse effects with the use of multiple agents as well as possible unwarranted interaction that can occur between the two agents.

Single agent approach to dual tumor cell and endothelial cell compartment targeting has been explored with agents such as Vandatenib (ZD6474). However, recent clinical trials of Vandetanib in lung cancer has been disappointing [36]. Additionally, TKIs with dual specificity usually have target profile that preferentially inhibits one of their targets (ZD6474 is more than ten times selective for VEGFR2 than EGFR) and also have non-specific off target effects. The STAT3 decoy oligonucleotide has greater specificity as it is an oligonucleotide, and we have shown previously that it has very low toxicity profile in primates [12]. Taken together with the previously demonstrated antitumor activity of this agent as well as anti-angiogenic effects demonstrated in this study, we believe that the STAT3 decoy oligonucleotide represents a promising single agent approach to targeting both the tumor and vascular compartments in various malignancies. Lastly, the elucidation of antiangiogenic properties of STAT3 decoy oligonucleotide elucidated in this study will aid in the identification of relevant biomarkers in clinical trials of STAT3 inhibitors and should allow for proper patient selection for these trials.
